# The Potential Application of Raw Cadmium Sulfide Nanoparticles as CT Photographic Developer

**DOI:** 10.1186/s11671-016-1424-7

**Published:** 2016-04-28

**Authors:** Qiang Wu, Lingxin Huang, Zhan Li, Wenzhen An, Dan Liu, Jin Lin, Longlong Tian, Xinling Wang, Bo Liu, Wei Qi, Wangsuo Wu

**Affiliations:** Radiochemical Laboratory, Lanzhou University, Lanzhou, 730000 China; Lanzhou Institute of Chemistry Physics, Chinese Academy of Science, Lanzhou, 730000 China; School of Life Sciences, Lanzhou University, Lanzhou, 730000 China; College of Pharmacy, Xinjiang Medical University, Urumqi, 830011 China; Huazhong University of Science and Technology, Wuhan, 430074 China

**Keywords:** Biodistribution, Damage, CdS, Mice

## Abstract

With the development of science and technology, new applications about nanoparticles should be explored to achieve full-scale knowledge. Therefore, in this work, the toxicity and potential application of raw cadmium sulfide nanoparticles (CdS) in vivo were further studied through ICP-OES and CTs. Surprisingly, CdS exhibited an excellent photographic property, except for finding the accumulation of CdS in the lungs, liver, spleen, and kidney with a strong dependence on time; it is also found that there were a significant uptake in the pancreas for an obvious CT imaging. And the following investigations showed that the raw CdS could damage the tissues accumulating nanoparticles. Through this work, it can be seen that the raw CdS being modified might be an excellent photographic developer for detecting cancers or other diseases.

## Background

Cadmium sulfide nanoparticles (CdS), especially their quantum dots (QDs) have been raised a great deal of attention as a special class of nanoparticles (NPs), due to their fluorescence and semiconductor properties [1]. At the same time, for their excellent luminescence, continuous excitation spectrum, controllable and narrow emission bands, and ease of the functionalization for tissue targeting, they show a great promise for medical imaging and treatment of disease [2]. However, before those nanoparticles are applied in medical field widely, the safety assessment of biology and environment needs to be investigated detailedly. Thus, a large amount of investigations on toxicity of CdS have been carried out for this purpose in vivo and in vitro. Buffet et al. [3] examined the toxicity effects of CdS-engineered nanoparticles compared to soluble Cd, on marine ragworms exposed for 14 days to these contaminants (10 μg Cd L^−1^) in seawater or via food, and they pointed out oxidative processes as the main consequences of exposure to Cd-based NPs in worms. Domingos et al. [4] found that CdS exerted higher toxicity compared to the same amounts of soluble Cd on bacteria and algae, suggesting a specific nanoeffect. King-Heiden et al. [5] also indicated that CdS also produced distinctly different toxicity that could not be explained by Cd release. Using Cd^2+^ ions, they found that zebrafish larvae showed clear signs of Cd toxicity. However, nanoparticles were even more potent and produced end points of toxicity distinct from that of Cd^2+^. But it was also reported that the cadmium from the degradation of CdS could be redistributed over time. Yang et al. [6] and Rzigalinski et al. [7] have reviewed early mouse studies of toxicity of Cd-based QDs, which all showed the absence of any significant toxicity at low dosage.

It is reported that the primary mechanism for CdS cytotoxicity was introduction of free radical formation [8, 9]. Active cadmium-based QDs core did participate in radical formation. At the cellular level, cadmium induced oxidative stress by depletion of endogenous antioxidants such as glutathione and was associated with mitochondrial damage, induction of apoptosis, and disruption of intracellular calcium signaling [10]. Where HepG2 cells were treated with different concentrations of Cd, a rapid and transient ROS generation had triggered Cd-induced apoptosis. Luo et al. [11] suggested that cadmium-containing QDs caused an increase of intracellular ROS levels in mouse renal adenocarcinoma (RAG) cells and induced autophagy and subsequent apoptosis. Li et al. [12] elucidated the relationship between Cd^2+^ and oxidative stress with experiments of biosurfactant-stabilized CdS (bs-CdS). They observed that bs-CdS had the capacity to generate free radicals indirectly and induced oxidative stress and apoptosis by releasing Cd^2+^ in cells. Singh et al. [13] synthesized highly stable and surface-protected CdS induced apoptotic cell death by selectively generating excess ROS in human prostate cancer lymph node carcinoma of the prostate (LNCaP) cells.

As well known, clinically, the frequently used CT photographic developer was iodine-containing compounds for the high density and low toxicity of iodine. But most of the iodine-containing compounds existed various defects, such as difficult to synthesize, non-targeting, and rapid metabolism. And so, people made a lot of exploratory development by using carbon nanoparticles to seek new photographic materials [14–16], but there also a problem that some special ions or groups must be linked to the surface of nanoparticles to achieve image effect in vivo. With a band gap of 2.4 eV and high electron mobility, CdS has high photocatalytic reactions and photoenergy conversion efficiency. And so, CdS has been generally considered as a strong candidate for high efficiency visible-light-driven photocatalysts [17]. As the same mechanism for CT photographic developer, CdS nanoparticles may be an excellent CT photographic developer. However, there are rare reports about the potential application of CdS nanoparticles in CT photographic developer. Therefore, in this experiment, the raw cadmium sulfide nanoparticles (CdS) are injected intravenously to the mice to determinate the property of photographic development by CT, and also to evaluate their acute toxicological and pathological effects in vivo. Through this work, it can provide basic data in vivo of CdS to help doctors alleviate the negative effects of Cd-containing nanoparticles and facilitate comprehensive utility of Cd-containing nanoparticles in treatment and diagnosis of disease.

## Methods

### Materials

The raw CdS were purchased from Shanghai Biological Technology Co., Ltd, with particle size about 1–30 nm. And the CdS were characterized by XRD (D5000, Siemens, Germany), Jeol2010 TEM (Jeol, USA), TGA, and Raman spectrum Luminescence spectrometer (LS55, Perkin Elmer, USA). At the same time, the UV-visible spectrum of CdS nanoparticles was recorded with a Perkin-Elmer Lambda 25 spectrophotometer. And the CdS were prepared 2.5 g/L suspension by PBS. All chemical reagents were analytically pure unless specified otherwise.

### Methods

#### Biodistribution of CdS in Mice

Kunming mice (female to male = 1:1) initially weighing 15 to 18 g were provided by Laboratory Center for Medical Science, Lanzhou University, Gansu, China. All animals were housed in individual cages in a temperature (21 to 22 °C) and light (from 0800 to 2000 h) controlled environment and were fed food and tap water ad libitum. All animal protocols were in accordance with the European Communities Council Directive of November 24, 1986 (86/609/EEC), and approved by Institutional Animal Care and Use Committees of Gansu Province Medical Animal Center and Lanzhou University Animal Committees Guideline. All mice (about 40 mice) were injected intravenously about 400 μg/mouse CdS solution. And the exposure groups of mice (six mice) were sacrificed at 1, 6, 16, 24, and 48 h, respectively, and then the blood (1 mL), heart, liver, spleen, lungs, and kidney were harvested and weighed. Then the selected tissues were digested and diluted to a certain concentration [18]. At last, the Cd content of the solution was measured through ICP-OES (ICP3000). Moreover, the mice after exposure CdS about 2 and 6 h were provided to CT with dual-energy spectral CT imaging mode (CT from Lanzhou University NO2 Hospital, Discovery CT 750HD, GE healthcare). The GSI scan parameters were as follows: Prep Group,30 s; scan mode, axial; gantry rotation speed, 0.5 s/circle; tube voltage, 140 and 80 kV; tube current, 630 mA s; detector coverage,20 mm;30 %ASIR; matrix size,512; slice thickness, 0.625 mm. Through those experiments, the biodistribution and photographic property of CdS in mice were determined, and the detailed information was exhibited as the “Result and Discussion” section.

#### The Exposure Dosage Effect on Biochemical Indexes

The mice (six mice/group) were exposed to 0, 100, 200, and 400 μg/mouse CdS solution, respectively. Then, the exposure mice were sacrificed at 24 h later, and the blood was collected to obtain serum. The collected blood was stayed in room temperature about 15 min, and then were centrifuged at 4000 rpm about 10 min, the supernatant (serum) were collected and kept in 4 °C refrigerator. At the next day, the blood urea nitrogen (BUN), creatinine (CREA), cystatin-C (Cys-C), alanine aminotransferase (ALT), aspartate aminotransferase (AST), and total bilirubin (TB) contents in serum were measured by ELISA kit (purchasing from Elabscience Biotechnology Co., Ltd). Through the above experiment, the effect of the exposure dosage of CdS on biochemical indexes were investigated.

#### Histopathology

The exposure groups of mice were sacrificed at about 24 h, and tissues such as the heart, liver, spleen, lungs, kidney, and pancreas were collected and the fixed right lobe from animals in each group was embedded in paraffin, sectioned onto slides, and stained with hematoxylin and eosin (H&E). H&E-stained slides were qualitatively analyzed for indications of inflammation and injury by a certified veterinary pathologist who was blinded to the treatment groups.

## Results and Discussion

### Materials Characterization

**Fig. 1:**
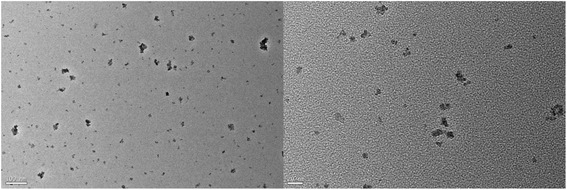
The TEM of CdS

**Fig. 2:**
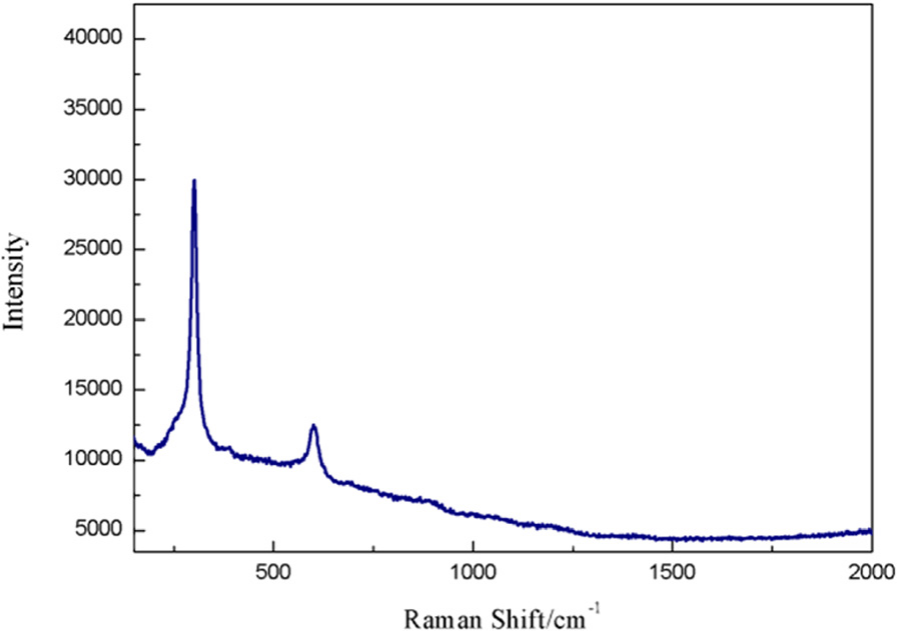
The Raman spectrum of CdS

**Fig. 3:**
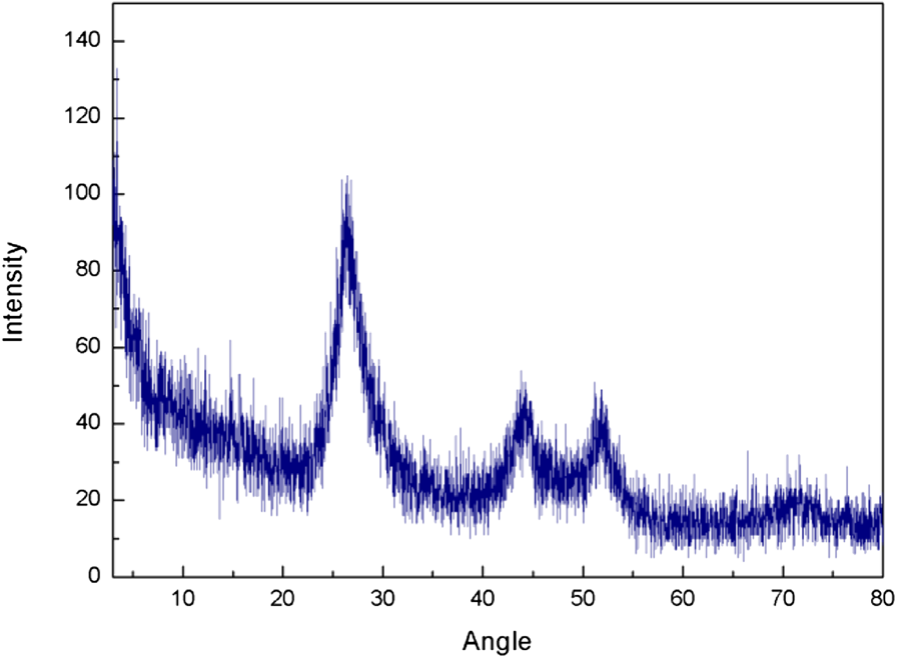
The XRD characterization of CdS

**Fig. 4:**
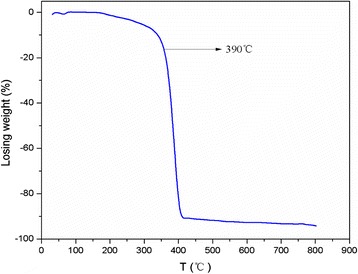
The TGA of CdS

The raw CdS were provided to the characterization of TEM, Raman spectrum, and XRD, and the results as Figs. 1, 2, 3, and 4. The TEM results of CdS showed that the nanoparticles size of CdS was about 30 nm with spherical particle state, which agrees with previous results [19, 20]. Figure 2 shows the result of Raman spectrum of CdS had the absorption peaks at 285 and 585 cm^−1^, respectively, indicating the characteristic absorption peaks of CdS nanoparticles [21–23]. Figure 3 shows the XRD pattern of a typical CdS nanoparticles sample. The XRD peaks are very broad, indicating the very fine size of the sample grains. The XRD pattern exhibited prominent broad peaks at 2*θ* values of 26.5°, 43.96°, and 52.13°, which could be indexed as scattering from the (111), (220), and (311) cubic phase CdS planes, respectively, according to JCPDS file NO.10–454. By using the Scherrer’s equation *d* = 0.8*λ*/*β*cos*θ*, where *λ* is the wavelength of the X-ray radiation, *β* is the full width at half maximum (FWHM) of the (111) peak, and *θ* is the angle of diffraction, the average size of the CdS nanoparticles was determined to be of the order of 3 nm. The only one losing weight peak of Fig. 4 showed that the purity of raw CdS was very high.

### Biodistribution of CdS in Mice

**Fig. 5:**
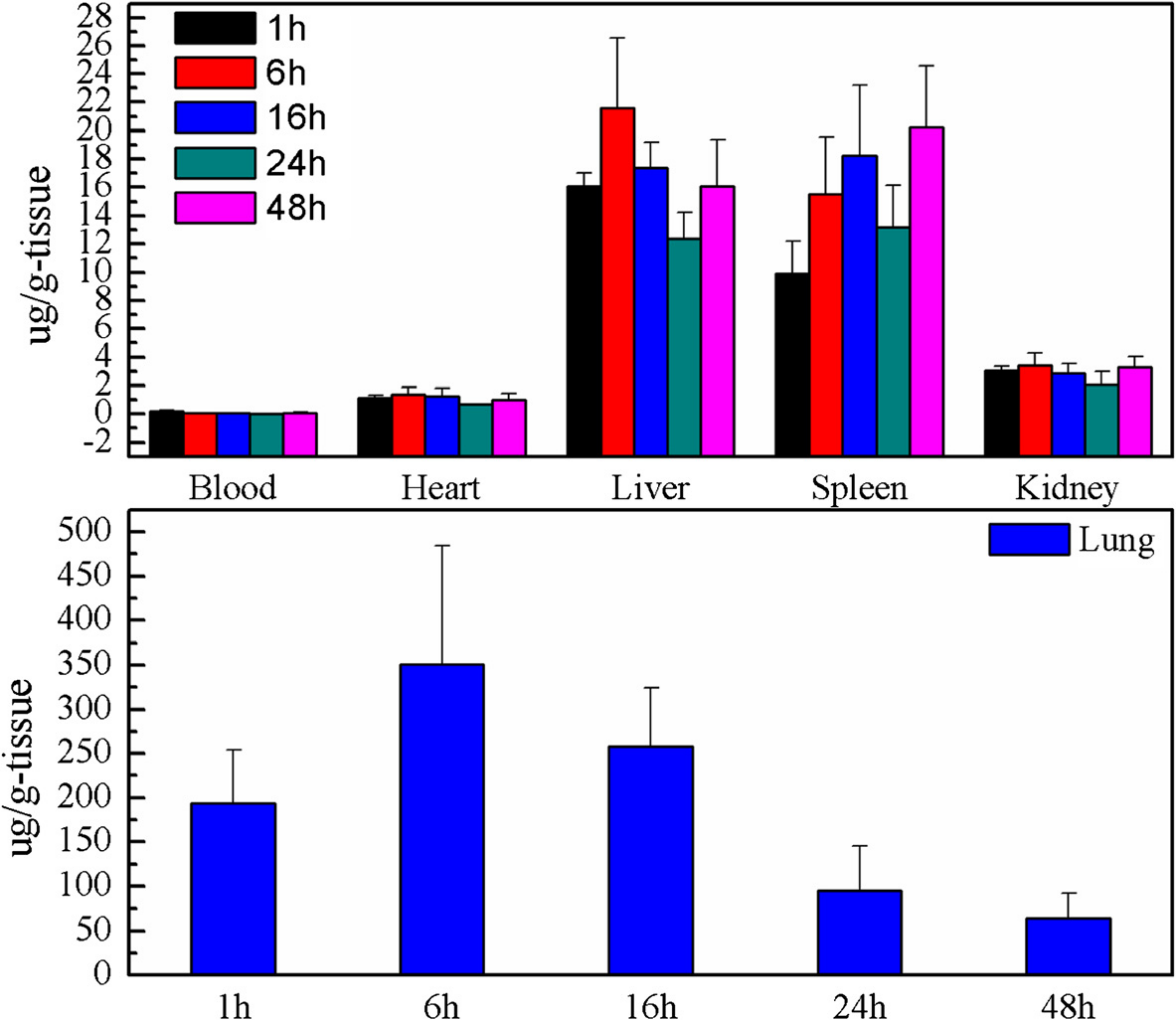
The tissue biodistribution of CdS at 1, 6, 16, 24, and 48 h after exposure of CdS in mice (*n* = 6, ±SEM)

**Fig. 6:**
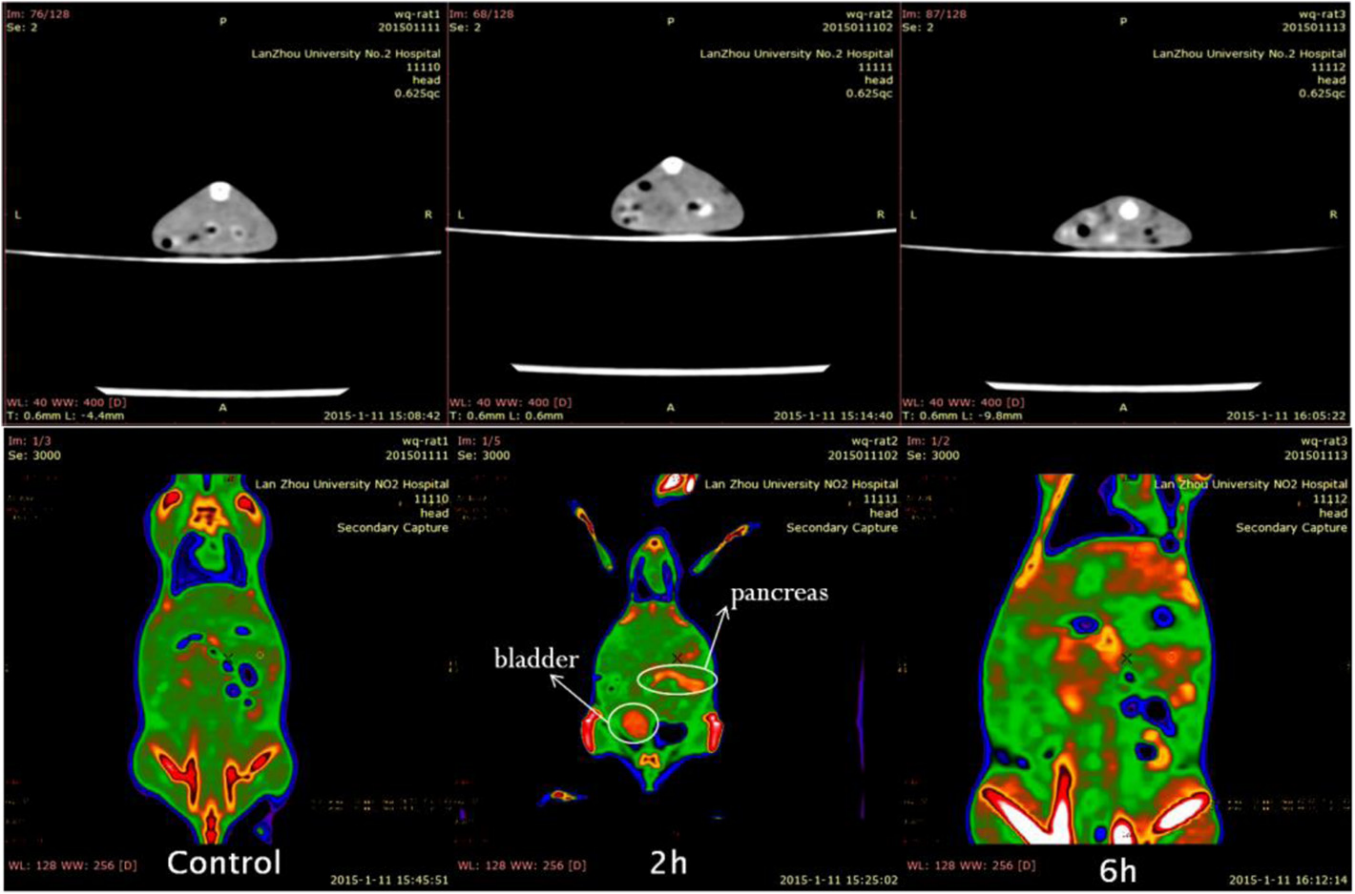
The CT imaging of CdS after exposure at 2 and 6 h in mice

It could be seen from Fig. 5 that the tissues biodistribution of CdS after exposure to mice could change with time passing. The results indicated that most of CdS were retained by the lungs after injection intravenously to mice, and the tissues of the liver, spleen, and kidney had also a certain degree of uptake; the largest accumulation were got at 6 h for the heart, liver, lungs, and kidney tissues (Fig. 5). At the same time, the CdS accumulated in tissues could decrease gradually with time going except for that in the spleen, but increased in the heart, liver, spleen, and kidney at 48 h after exposure (Fig. 5). It was reported that nanoparticles injected intravenously into the blood would pass through the right atrium, right ventricle, lungs, left atrium, and into the left ventricle successively [18]. In the left ventricle, nanoparticles would be pumped into the blood circulation and carried into every tissue. In this process, nanoparticles and other mechanism materials would be captured by the pulmonary capillary bed to protect heart from being hurt. Therefore, there was a largest CdS accumulation in the lungs after injection intravenously into mice. From the characterization of CdS (Figs. 1, 2, 3 and 4), it could be seen that the average size of CdS were very small, just 3 nm. Thus, the part of CdS could pass through the pulmonary capillary bed and enter into the blood circulation, and then into other tissues, and so, the CdS had the largest accumulation and then rapidly decreased in the lungs after 6 h (Fig. 5). It was reported that the high-level accumulation of nanoparticles in the organs depended on the rapid capture of the reticuloendothelial system (RES), and RES capture occurred via opsonization, i.e., opsonins binding to nanoparticles in the plasma via recognition by phagocytes in the RES [24, 25]. As well known, the liver and spleen were the immune organs of biology body with a lot of macrophages (e.g., Kupffer cells); hence, CdS as the invasive materials for biology body were captured by RES in the liver and spleen with a mass of phagocytes, resulting in high uptake of the liver and spleen (Fig. 5). In addition, the spleen was the largest immune organ of biology body, and had more lymphocytes and macrophages, so the accumulation of CdS in the spleen increased after exposure. The accumulation of the kidney showed that the CdS could be excreted through the urinary system (Figs. 5 and 6), and so the content of CdS in tissues decreased with time extension. However, the accumulation of CdS increased in the heart, liver, spleen, and kidney 48 h after exposure, it might be attributed to the redistribution of CdS from the lung tissues or the releasing of Cd^2+^ from the degradation of CdS nanoparticles [6], but this speculation needs to be further studied through experiments.

However, it was interesting that the CT imaging of CdS showed an obvious absorption in the lungs, liver, spleen, kidney, and bladder, especially in the pancreas (Fig. 6), as we know, the phenomenon about the presence of nanoparticles in the pancreas was found firstly in this work. Therefore, it should be discussed how CdS could enter into the pancreas. As early as 1901 year, Opie et al. reported the common-channel hypothesis as the potential triggering mechanism for gallstone-induced pancreatitis [26]. They found the pancreatic duct and the common bile duct communicated, and called this communication as a common channel, which could have allowed for the bile to enter into the pancreas. Accordingly, they proposed that the reflux of the bile through the common channel into the pancreatic duct represents the triggering event for biliary pancreatitis. What is more, it was reported that the gallstone could stimulate Oddi’s sphincter during rolling into the duodenum leading to congestion, edema, and spasm, and then resulted in the function disorder of Oddi’s sphincter, so far as to reverse shrink, which could produce bile or duodenum content regurgitation [27]. Therefore, authors thought that CdS could enter into and damage the liver after exposure to mice, and parts of CdS might enter into the bile duct with bile, and then further into the duodenum. In this process, the CdS stimulated Oddi’s sphincter and triggered function disorder, resulting the CdS enter into the pancreas with bile or duodenum content regurgitation into the pancreas. And so, there was a high uptake of CdS in the pancreas (Fig. 6).

### The Exposure Dosage Effect on Biochemical Indexes

**Fig. 7:**
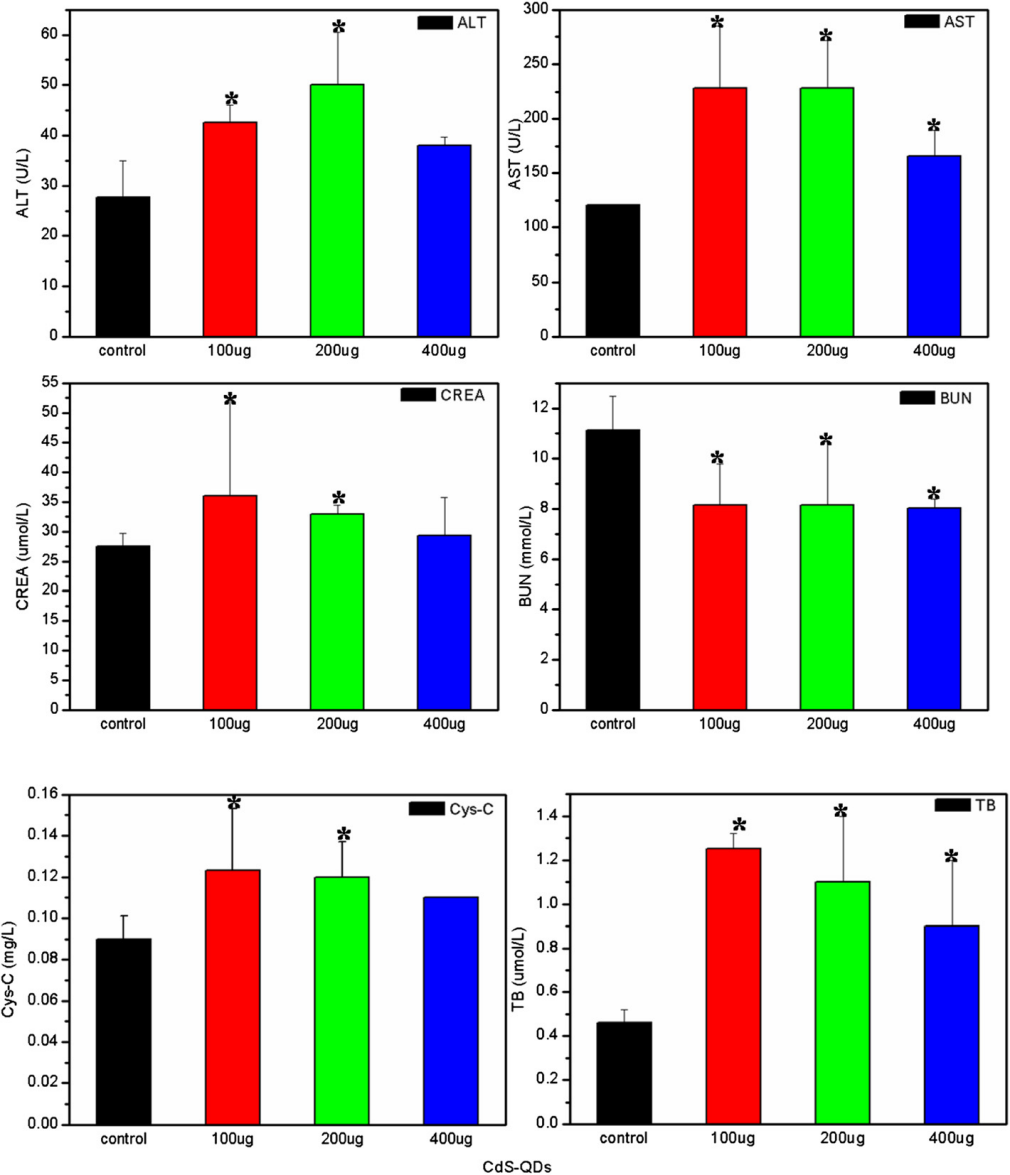
The changes of biochemical index content in serum after exposure of CdS to mice intravenously (*p < 0.05 for the groups vs. control group, *n* = 6, ±SEM)

The exposure dosage of CdS effecting on the biochemical indexes in serum was studied, and such as the BUN, CREA, Cys-C, ALT, AST, and TB contents in serum were measured through ELISA kit. As the results shown of Fig. 7, compared with the contents of biochemical indexes of control group, there was a significant difference from that of exposure groups (*p < 0.05). The ALT, AST, and TB contents of the exposure groups were much higher than that of the control group, indicating that the CdS had a serious damage on the liver. But the CREA, Cys-C, and BUN contents were abnormal in serum, which might show that the kidney was injured after exposure of CdS to mice (Fig. 7). Moreover, it was also showed that the changes of biochemical indexes in serum depended on the exposure dosage of CdS. The effect levels decreased slightly with the exposure dosage increased from 100 to 400 μg/mouse, but when the exposure dosage got to 400 μg/mouse, the tissue damages decreased slightly compared with that exposure to low dosages. The author inferred that the reason could be relative to the agglomeration of CdS nanoparticles in the solution (Fig. 1).

### Histopathology

**Fig. 8:**
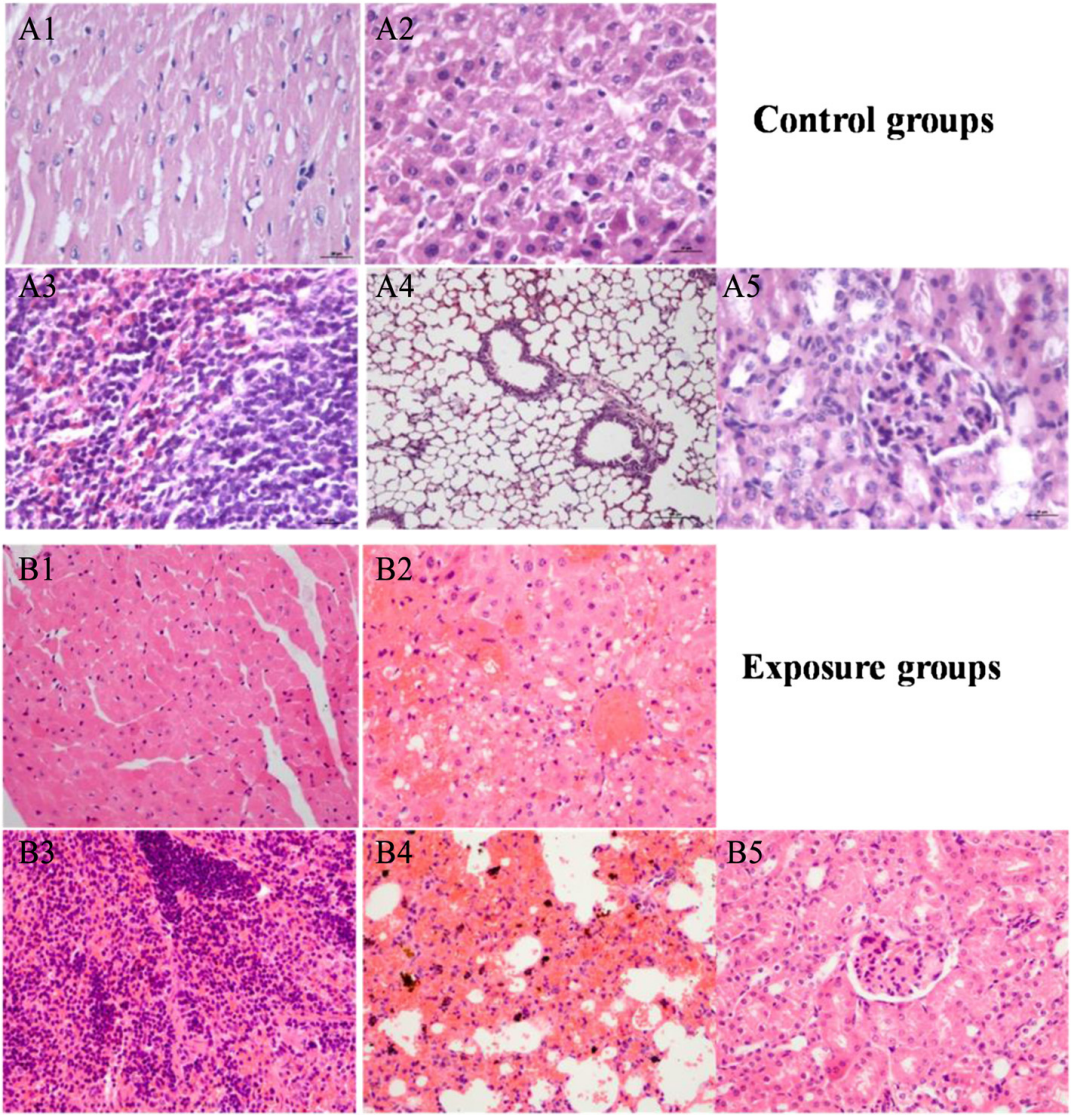
The histopathology (×400) of tissues after exposure CdS to mice (*A1–A5* and *B1–B5* for the heart, liver, spleen, lung, and kidney, respectively)

**Fig. 9:**
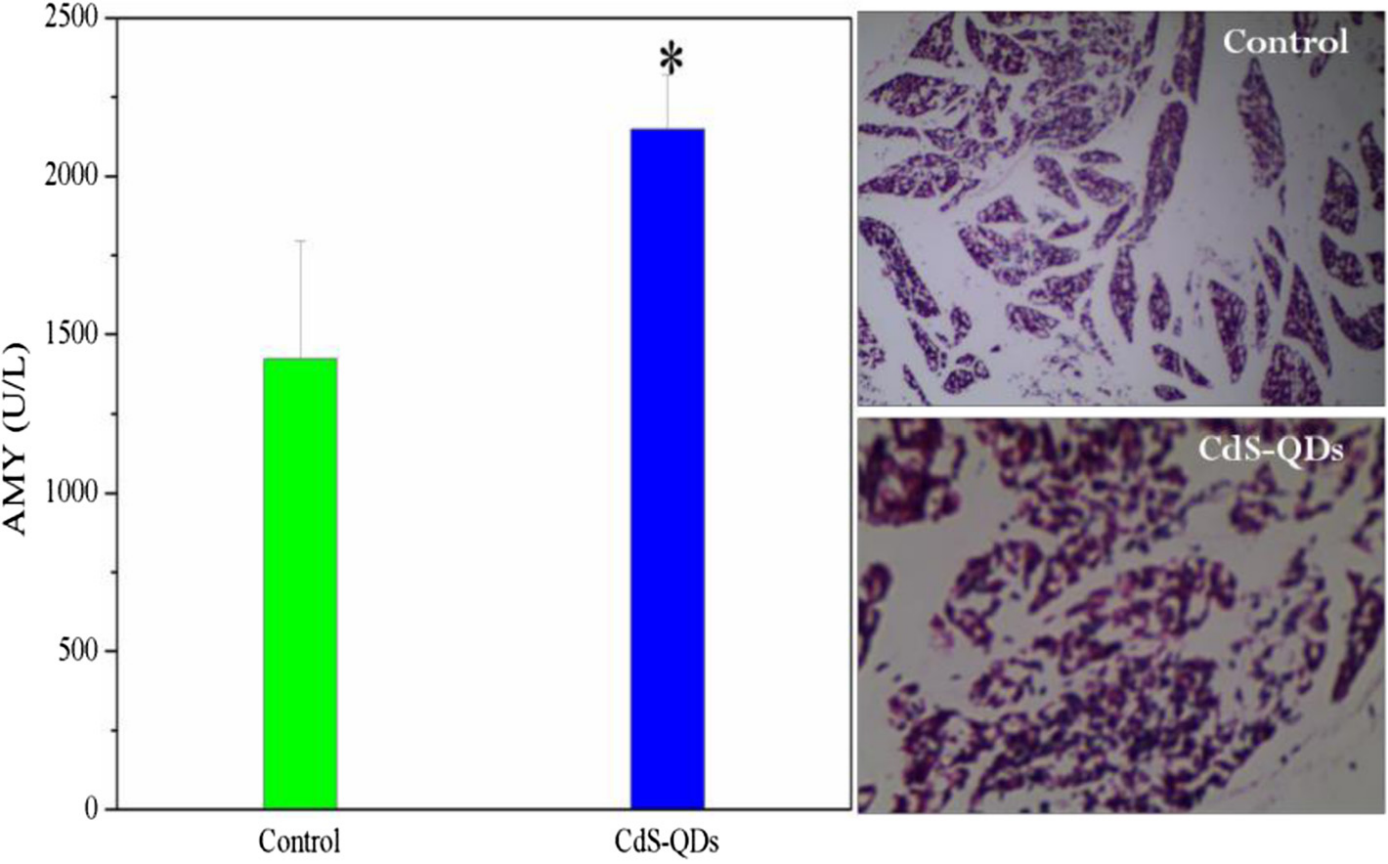
The changes of AMY content in serum and histopathology of the pancreas after exposure CdS to mice (*p < 0.05 for the groups vs. control group, *n* = 6, ±SEM)

The sections of organic tissues were cut to perform microscopic examination, and shown in Fig. 8. The results showed that the CdS nanoparticles could cause the extensive injury on tissues containing the liver, spleen, kidney, and lung, but the control groups of normal saline was normal. The cell lineage disorder, hepatic lobules disappeared, and hydropic degeneration with focal inflammation could be observed from the liver section, and there was a severe hemorrhage phenomenon. The splenic sinus eclasis, size disorder of follicular, hyperplasia of extramedullary hematopoietic giant cells, and serious hemorrhage phenomenon could be seen from the spleen section. The section of the lung tissue showed bronchial epithelial disorder and alveolar walls broken with a severe bleeding, in addition, the brown stain of section indicated that there were a lot of nanoparticles in the lung tissues. The lesion of the kidney could also be observed with glomerular swelling, smaller glomerular capsular, and mesangial cell proliferation. Figure 9 indicates that the amylase (AMY) content in serum of the exposure group decreased compared with that of the control group, and the histology section of the pancreas also exhibited pathological changes. Those pathological measurement results were according to the biochemical indexes content changes in serum. Therefore, it could be seen that the CdS damaged indeed the tissues of the mice after being exposed.

## Conclusions

From this work, it could be seen that the CdS exhibited an excellent property of CT photographic developer with a certain toxicity in vivo. CdS were mainly retained in the lungs, but slight in the liver, spleen, and kidney, with a strong dependence on time. In addition, the accumulation of CdS in the pancreas was found firstly, and authors gave a detailed discussion about this point. Accordingly, the biochemical indexes and histology sections also indicated that the CdS had caused serious damages to the tissues. Through this work, it could help doctors alleviate the negative effects of Cd-containing nanoparticles and facilitate comprehensive utility of Cd-containing nanoparticles in medicine.
